# P42 Pattern of antibiotics resistance and phenotypic characterization of MDR bacteria isolates in four hospitals of Littoral Region, Cameroon

**DOI:** 10.1093/jacamr/dlac004.041

**Published:** 2022-02-16

**Authors:** Merlin Jonas Wandji Takemegni, Clement Jules Nguedia Assob, Jérôme Ateudjieu, George Orock Enow, Francois-Xavier Mbopi-Keou, Marcelin Ngowe Ngowe

**Affiliations:** Department of Medical Laboratory Sciences, New-Bell District Hospital, Douala, Cameroon; Department of Medical Laboratory Sciences, Faculty of Health Sciences, University of Buea, Buea, Cameroon; Department of Medical Laboratory Sciences, Faculty of Health Sciences, University of Buea, Buea, Cameroon; Department of Biological Sciences, Faculty of Medicine and pharmaceutical Sciences, University of Douala, Douala, Cameroon; Division of Health Research, M. A. SANTE (Meilleur Accès aux Soins de Santé/Better Access to Health Care), Yaoundé, Cameroon; Department of Public Health, University of Dschang, Dschang, Cameroon; Division of Health Operations Research, Ministry of Public Health, Yaoundé, Cameroon; Department of Medical Laboratory Sciences, Faculty of Health Sciences, University of Buea, Buea, Cameroon; Department of Microbiology, Virology and Immunology, Faculty of Medicine and Biomedical Sciences, University of Yaoundé I, Yaoundé, Cameroon; Department of Biological Sciences, Faculty of Medicine and pharmaceutical Sciences, University of Douala, Douala, Cameroon; Department of Surgical Sciences, Faculty of Medicine and pharmaceutical Sciences, University of Douala, Douala, Cameroon

## Abstract

**Background:**

Contaminated hospital surface have been recognized to be the most significant reservoir of MDR bacteria (MDRB). The aim of this study was to describe phenotypical characteristics of MDRB species surface contaminants in four hospitals of Littoral region, Cameroon.

**Methods:**

We conducted a descriptive hospital-based cross-sectional study from December 2018 to May 2019. A simple random sampling was used to swab 10 selected equipment items and 10 materials in the mornings after disinfection but before the start of work in seven units (Medical, Paediatric, Operating Theatre, Laboratory, Surgical, Emergency and Maternity). After inoculation in four agar media consecutively (eosin methylene blue, CLED, mannitol salt agar and blood agar) and incubation in appropriate conditions, the Kirby–Bauer disc-diffusion method was used for antimicrobial susceptibility testing. Phenotypic methods using specific indicator discs were used for screening and confirmation of MDRB, of which: ESBL-producing, MRSA, vancomycin-resistant *Staphylococcus aureus* (VRSA), vancomycin-resistant *Enterococcus faecalis* (VRE),vancomycin-resistant CoNS (VRCoNS), MDR and XDR. Control strains, *Escherichia coli* ATCC 25922 (non ESBL-producer) and *Klebsiella pneumoniae* 700603 (ESBL-producer) and *S. aureus* ATCC 25923 (MSSA) were used to ensure ability to support growth of the target organism(s), ability to produce appropriate biochemical reactions and adequate inhibition zone diameters.

**Results:**

Among 50.4% (119/236) showed positive bacteria growth, a total of 89 (13 species), predominant bacteria and those more likely to cause nosocomial infections were selected and tested each one with 18 antibiotics. There was high level of resistance to penicillin [amoxicillin (77.5%) and oxacillin (76.4%)], followed by 3G cephalosporin [ceftazidime (74.2%)] and monobactam [aztreonam (70.8%)]. Although the least level of resistance was observed in carbapenem [imipenem (5.6%)]. The overall prevalence of MDRB was 62.9% (56/89). MRSA were the most detected 57.5% (30/89), followed by ESBL 10.1% (9/89). The lowest percentage was recorded by VRE and XDR both with 1.1% (1/89). According to the type of MDRB in selected isolated bacteria, all the strains (100%) of two species, *Aeromonas hydrophila* and *Enterococcus faecalis*, were characterized MDRB. However, *S. aureus* strains reported significant rate of MDRB, 84.4% (38/45), and recorded 4.4% (2/45) strains of ESBL. Moreover, non-fermenting Gram-negative bacilli, *Pasteurella pneumotropica* provided 75% (3/4), as well as being the only Gram-negative bacilli species where one strain was resistant to more than three different class of tested antibiotics including carbapenems (imipenem) and was consequently named XDR with 25% (1/4). Military hospital of Douala and Emergency unit were the dominantly MDRB contaminated areas: 39.3% (22/56) and 17.9% (10/56), respectively.

**Conclusions:**

MDRB are a current public health problem and hospital surfaces are a worrying reservoir that can be spread to patients, health professionals and visitors. Our results could serve as a timely regional data of hospital surface epidemiological surveillance basis on which preventive strategy of HAIs and AMR should be built.
